# Proposal of a cutaneous lupus erythematosus‐like keratinocyte model in vitro under local conditions using interferon‐alpha and Poly I:C and its use in examining the therapeutic effects of tyrosine kinase 2 inhibitor

**DOI:** 10.1111/1346-8138.17318

**Published:** 2024-06-03

**Authors:** Yoko Kuba‐Fuyuno, Makiko Kido‐Nakahara, Gaku Tsuji, Sawako Sakai, Takeshi Nakahara

**Affiliations:** ^1^ Department of Dermatology, Graduate School of Medical Sciences Kyushu University Higashiku, Fukuoka Japan; ^2^ Research and Clinical Center for Yusho and Dioxin Kyushu University Hospital Higashiku, Fukuoka Japan

**Keywords:** CLE, deucravacitinib, IFNα, keratinocyte, Poly I:C

Lupus erythematosus is an autoimmune disease that has a broad spectrum of manifestations from skin lesions [cutaneous lupus erythematosus (CLE)] to impacts on various organs [systemic lupus erythematosus (SLE)]. Despite expansion of the treatment options for SLE, including targeted therapies, there is still an unmet need for novel therapies that effectively control CLE.^1^

To facilitate the novel development of therapies for CLE, lupus mouse models or skin samples biopsied from CLE patients can be used.^2^ However, in vivo experiments with mice are costly and laborious, and the use of patient specimens involves certain ethical issues and is invasive. In view of this, it would be beneficial to develop an in vitro lupus‐like keratinocyte model to promote the development of new therapies for CLE.

Polyinosinic‐polycytidylic acid (Poly I:C), a synthetic nucleic acid, has been reported to accelerate the emergence of lupus manifestations in several lupus‐prone mouse models in macrophages and kidney cells via the stimulation of toll‐like receptor 3.^3^ In a mouse model of impaired clearance of endogenous nucleic acids, SLE‐like skin symptoms have been reported.^4^ Moreover, one crucial hallmark of SLE is the dysregulation of type 1 interferon (IFN), and the approval of anifrolumab, an anti‐type 1 IFN receptor antibody, marks a major advance in the treatment of SLE.^1^ In this study, we aimed to establish a CLE‐like keratinocyte model by stimulating normal human epidermal keratinocytes (NHEKs) with IFNα and Poly I:C.

Deucravacitinib, an oral, selective, allosteric inhibitor of tyrosine kinase 2 (TYK2), is a potential treatment for SLE.^5^ Although a phase 2 trial (PAISLEY) revealed that deucravacitinib was superior to placebo in reducing SLE disease activity and Cutaneous LE Disease Area and Severity Index (CLASI) 50,^5^ few reports on in vitro studies of its effects on keratinocytes have been published. We thus also examined whether deucravacitinib inhibits CLE‐like inflammation in IFNα‐ and Poly I:C‐stimulated NHEKs.

In the first step, we compared the characteristics of acute CLE (ACLE) in the skin of SLE patients (*n* = 7) and normal controls (*n* = 7) by analyzing the expression of IFNκ, CXCL10, and caspase7 in the epidermis immunohistochemically. All SLE patients met 1997 American College of Rheumatology revised criteria for the classification of SLE. All protocols were approved by the Institutional Review Board of the University of Kyushu Hospital. The results showed that IFNκ‐, CXCL10‐, and caspase7‐ positive keratinocytes were significantly increased in the epidermis of ACLE compared with the levels in normal skin (Figures 1a,b and Supporting Information S3a). Details of the purchased antibodies and reagents are provided in Supporting Information Tables S1 and S2.

**FIGURE 1:**
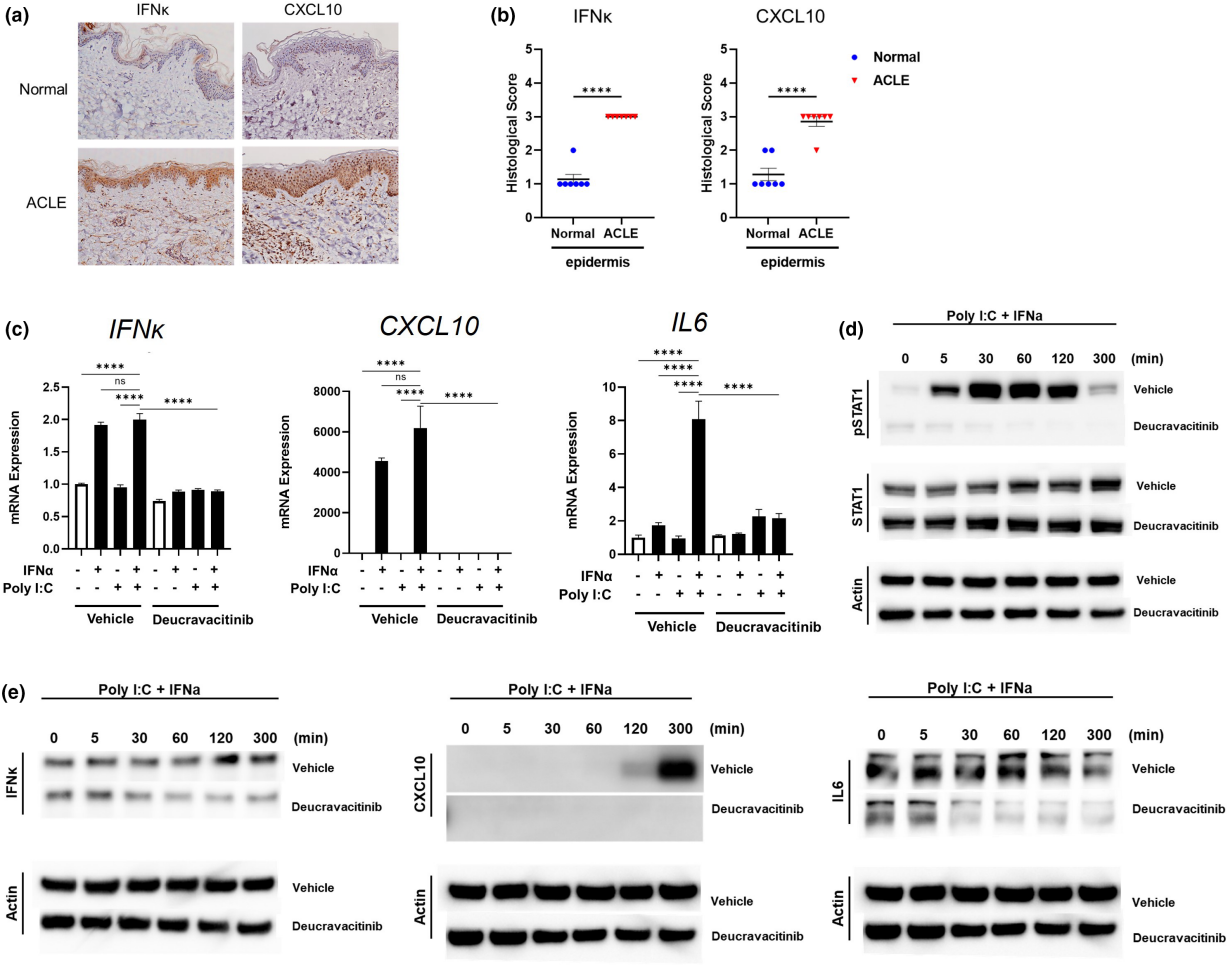
(a) IFNκ and CXCL10 immunostaining is stronger in the epidermis of acute CLE (ACLE) patients than in the epidermis of normal controls. Representative immunohistochemical analysis of IFNκ and CXCL10 expression in skin samples from ACLE patients (*n* = 7) and normal controls (*n* = 7). (b) Histological score of IFNκ and CXCL10 in the epidermis. ****P < 0.0001, unpaired Student's *t* test. (c) IFNα and Poly I:C significantly upregulated the expression of IFNκ, CXCL10, and IL6, which was blocked in the presence of deucravacitinib in cultured normal human keratinocytes (NHEKs). ****P < 0.0001, unpaired Student's *t* test. (d) IFNα and Poly I:C significantly increased pSTAT1 activity, which was inhibited by deucravacitinib in NHEKs. (e) The production of IFNκ, CXCL10, and IL6 proteins induced by IFNα and Poly I:C was also inhibited by deucravacitinib in NHEKs.

Next, we aimed to establish an in vitro CLE‐like keratinocyte model. To achieve this, NHEKs were stimulated with IFNα (15 ng/mL) and Poly I:C (15 ng/mL). The sequences of the primer pairs used for qRT‐PCR are shown in Supporting Information Table S3. The results revealed that NHEKs stimulated by IFNα and Poly I:C showed increased mRNA expression of IFNκ, CXCL10, and IL6 (*n* = 3) for 5 h (Figure 1c), and of B‐cell activating factor (BAFF), BAFF receptors [BAFF receptor, transmembrane activator and calcium modulator and cyclophilin ligand interactor (TACI)], caspase3, and caspase7 (*n* = 5), known as apoptotic signals, in NHEKs for 24 h (Supporting Information Figures S2a and S3b). Notably, IFNα alone upregulated them, but Poly I:C addition tended to increase their expression further. It is reported that IL6, BAFF, and BAFF receptors expression and apoptotic keratinocytes were increased in the epidermis in CLE.^6^, ^7^ These results indicate that stimulation of keratinocytes with IFNα and Poly I:C may serve as a CLE‐like keratinocyte model under localized conditions, as they exhibit a major CLE‐like signal confined to keratinocytes in vitro.

Next, the effect of deucravacitinib (10 μM) on these major CLE‐like signals was investigated by using it to treat NHEKs at the same time as IFNα and Poly I:C. On stimulation by IFNα and Poly I:C, pTYK2 and pSTAT1 levels were markedly upregulated in NHEKs, and deucravacitinib suppressed pTYK2 and pSTAT1 induced by IFNα and Poly I:C (Supporting Information Figures S1 and 1d). Notably, deucravacitinib suppressed IFNκ, CXCL10, IL6, and caspase3 and caspase7 mRNA and protein expression, and BAFF and BAFF receptor mRNA expression (Figures 1c,e and Supporting Information Figures S2a and S3b,c). IFNα and Poly I:C also induced inflammatory cytokines other than type I IFN, and cell death, which were suppressed by deucravacitinib (Supporting Information Figures S2b and S3d,e).

We believe that this localized keratinocyte model will be more useful in developing novel therapies for CLE, and the fact that deucravacitinib suppressed CLE‐like signal elevation by IFNα and Poly I:C is significant because the data support the effect of deucravacitinib identified in the PAISLEY phase 2 trial.^5^

## CONFLICT OF INTEREST STATEMENT

None declared.

## Supporting information


Supporting Information Figure S1.

Supporting Information Figure S2.

Supporting Information Figure S3.

Supporting Information Table S1.

Supporting Information Table S2.

Supporting Information Table S3.

## References

[1] Niebel D , de Vos L , Fetter T , Brägelmann C , Wenzel J . Cutaneous lupus erythematosus: an update on pathogenesis and future therapeutic directions. Am J Clin Dermatol. 2023;24:521–540. 37140884 10.1007/s40257-023-00774-8 PMC10157137

[2] Sarkar MK , Hile GA , Tsoi LC , Xing X , Liu J , Kahlenberg JM . Photosensitivity and type I IFN responses in cutaneous lupus are driven by epidermal‐derived interferon kappa. Ann Rheum Dis. 2018;77:1653–1664. 30021804 10.1136/annrheumdis-2018-213197 PMC6185784

[3] Kawato Y , Fukahori H , Nakamura K , Kubo K , Hiramitsu M , Morokata T , et al. Development of a novel Poly (I:C)‐induced murine model with accelerated lupus nephritis and examination of the therapeutic effects of mycophenolate mofetil and a cathepsin S inhibitor. Eur J Pharmacol. 2023;938:175440. 36463947 10.1016/j.ejphar.2022.175440

[4] Scholtissek B , Zahn S , Maier J , Klaeschen S , Braegelmann C , Wenzel J , et al. Immunostimulatory endogenous nucleic acids drive the lesional inflammation in cutaneous lupus erythematosus. J Invest Dermatol. 2017;137:1484–1492. 28351661 10.1016/j.jid.2017.03.018

[5] Morand E , Pike M , Merrill JT , van Vollenhoven R , Werth VP , Singhal S , et al. Deucravacitinib, a tyrosine kinase 2 inhibitor, in systemic lupus erythematosus: a phase II, randomized, double‐blind, placebo‐controlled trial. Arthritis Rheumatol. 2023;75:242–252. 36369798 10.1002/art.42391 PMC10100399

[6] Stannard JN , Reed TJ , Myers E , Lowe L , Sarkar MK , Kahlenberg JM , et al. Lupus skin is primed for IL‐6 inflammatory responses through a keratinocyte‐mediated autocrine type I interferon loop. J Invest Dermatol. 2017;137:115–122. 27646883 10.1016/j.jid.2016.09.008 PMC5183476

[7] Nürnberg W , Haas N , Schadendorf D , Czarnetzki BM . Interleukin‐6 expression in the skin of patients with lupus erythematosus. Exp Dermatol. 1995;4:52–57. 7757333 10.1111/j.1600-0625.1995.tb00222.x

