# Supplementary figures and images for: Proposal of a cutaneous lupus erythematosus‐like keratinocyte model in vitro under local conditions using interferon‐alpha and Poly I:C and its use in examining the therapeutic effects of tyrosine kinase 2 inhibitor
Source: J Dermatol. 2024 Jun 3;51(7):1031–3. doi: 10.1111/1346-8138.17318 (PMC11483919; doi:10.1111/1346-8138.17318)

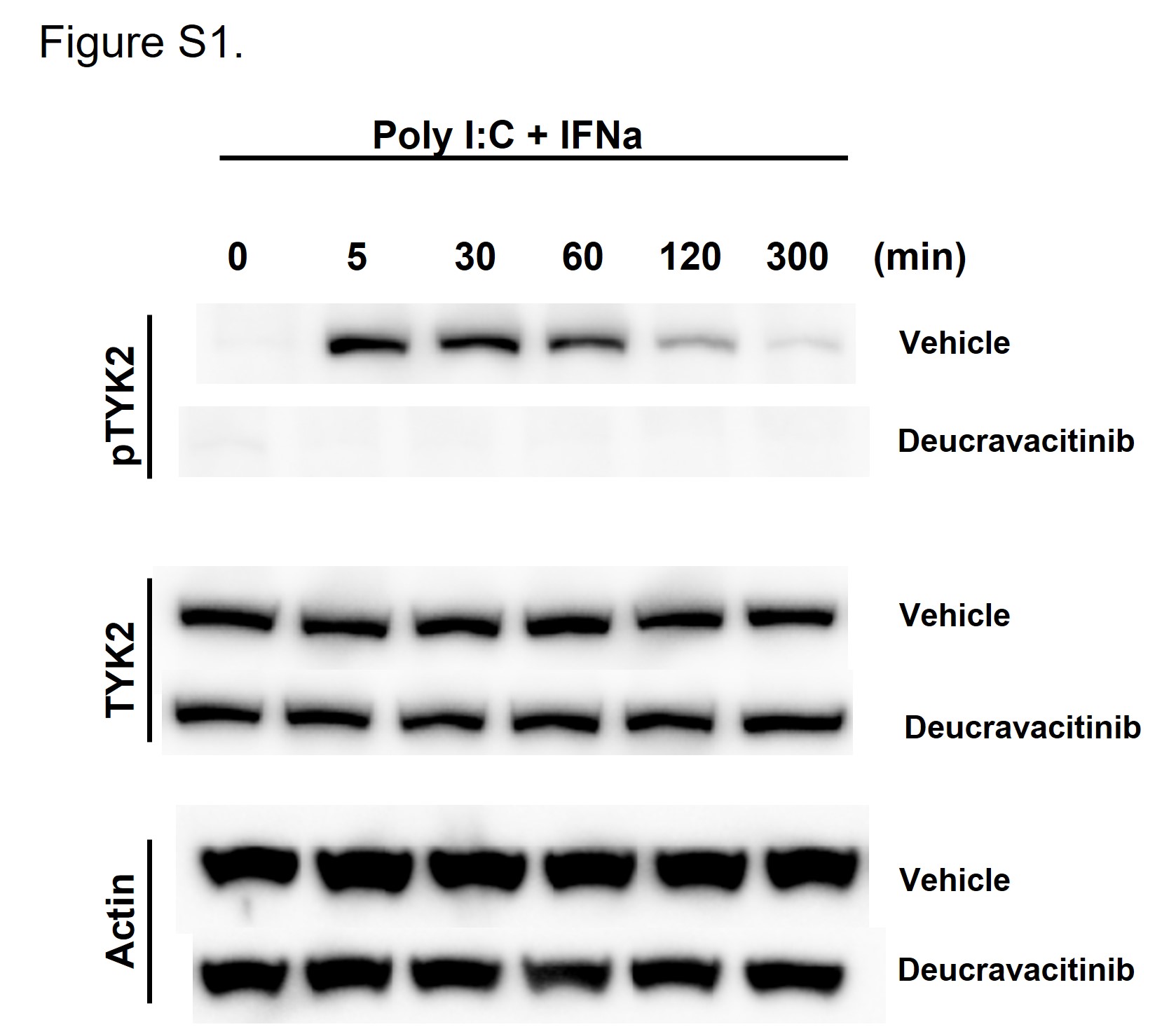

Supplement: Supplementary file 1 — Supporting Information Figure S1. [file JDE-51--s004.jpg]

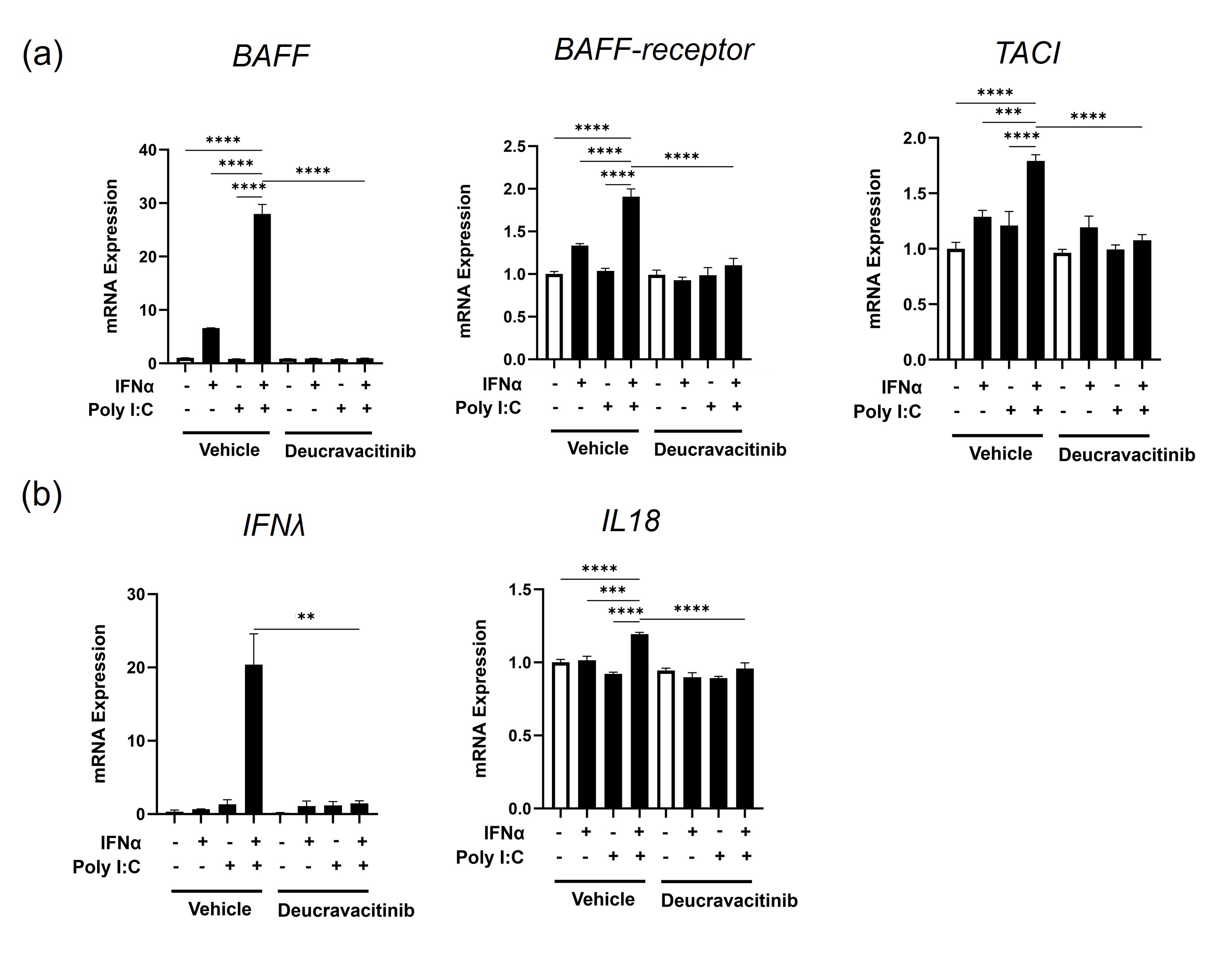

Supplement: Supplementary file 2 — Supporting Information Figure S2. [file JDE-51--s005.jpg]

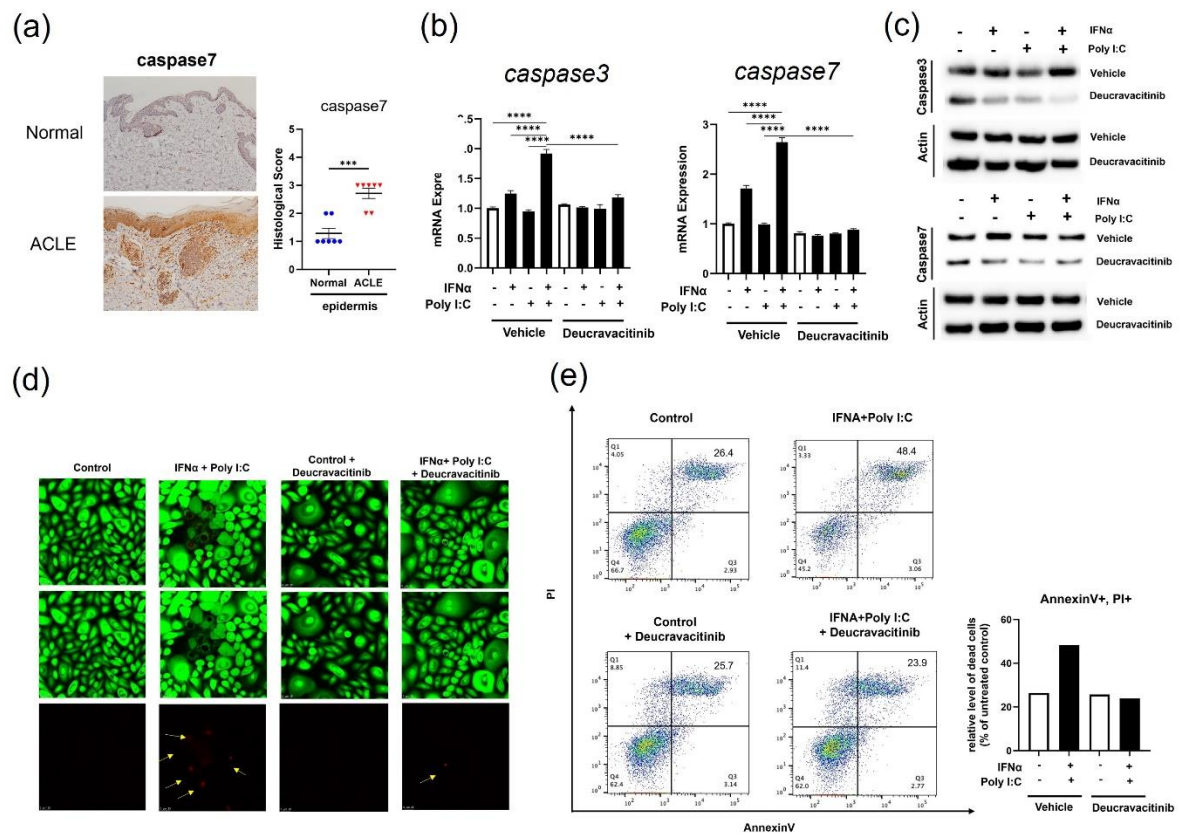

Figure S3

Supplement: Supplementary file 3 — Supporting Information Figure S3. [file JDE-51--s001.pdf]
